# A pandemic within a pandemic? Admission to COVID-19 wards in hospitals is associated with increased prevalence of antimicrobial resistance in two African settings

**DOI:** 10.1186/s12941-023-00575-1

**Published:** 2023-04-13

**Authors:** Linzy Elton, Muzamil Mahdi Abdel Hamid, John Tembo, Hana Elbadawi, Kwitaka Maluzi, Mohammed H. Abdelraheem, Teresa Cullip, Caren Kabanda, Kerry Roulston, Isobella Honeyborne, Margaret J Thomason, Kamal Elhag, Alaelddin Mohammed, Abdelsalam Adam, Kangwa Mulonga, Kapatiso Sikakena, Peter Matibula, Mwewa Kabaso, Ruth Nakazwe, Sombo Fwoloshi, Alimuddin Zumla, Timothy D McHugh

**Affiliations:** 1 Centre for Clinical Microbiology, University College London, London, UK; 2 Institute for Endemic Diseases, University of Khartoum, Khartoum, Sudan; 3 Sudan Atomic Energy Commission, Nuclear Application in Biological Sciences, Khartoum, Sudan; 4 HerpeZ, University Teaching Hospital, Lusaka, Zambia; 5 Institute for Global Health, University College London, London, UK; 6 MRC Clinical Trials Unit, University College London, London, UK; 7 Soba University Hospital, University of Khartoum, Khartoum, Sudan; 8 University Teaching Hospitals, Department of Internal Medicine, Infectious Diseases Unit, Lusaka, Zambia; 9 National Institute for Health and Care Research Biomedical Research Centre, University College London, London, UK

**Keywords:** COVID-19, SARS-CoV-2, Antimicrobial resistance, Infection prevention and control, Antimicrobial stewardship, Multi-drug resistance

## Abstract

**Background:**

Patients who develop severe illness due to COVID-19 are more likely to be admitted to hospital and acquire bacterial co-infections, therefore the WHO recommends empiric treatment with antibiotics. Few reports have addressed the impact of COVID-19 management on emergence of nosocomial antimicrobial resistance (AMR) in resource constrained settings. This study aimed to ascertain whether being admitted to a COVID-19 ward (with COVID-19 infection) compared to a non-COVID-19 ward (as a COVID-19 negative patient) was associated with a change in the prevalence of bacterial hospital acquired infection (HAI) species or resistance patterns, and whether there were differences in antimicrobial stewardship (AMS) and infection prevention and control (IPC) guidelines between COVID-19 and non-COVID-19 wards. The study was conducted in Sudan and Zambia, two resource constrained settings with differing country-wide responses to COVID-19.

**Methods:**

Patients suspected of having hospital acquired infections were recruited from COVID-19 wards and non-COVID-19 wards. Bacteria were isolated from clinical samples using culture and molecular methods and species identified. Phenotypic and genotypic resistance patterns were determined by antibiotic disc diffusion and whole genome sequencing. Infection prevention and control guidelines were analysed for COVID-19 and non-COVID-19 wards to identify potential differences.

**Results:**

109 and 66 isolates were collected from Sudan and Zambia respectively. Phenotypic testing revealed significantly more multi-drug resistant isolates on COVID-19 wards in both countries (Sudan *p* = 0.0087, Zambia *p* = 0.0154). The total number of patients with hospital acquired infections (both susceptible and resistant) increased significantly on COVID-19 wards in Sudan, but the opposite was observed in Zambia (both *p* = ≤ 0.0001). Genotypic analysis showed significantly more β-lactam genes per isolate on COVID-19 wards (Sudan *p* = 0.0192, Zambia *p* = ≤ 0.0001).

**Conclusions:**

Changes in hospital acquired infections and AMR patterns were seen in COVID-19 patients on COVID-19 wards compared to COVID-19 negative patients on non-COVID-19 wards in Sudan and Zambia. These are likely due to a potentially complex combination of causes, including patient factors, but differing emphases on infection prevention and control, and antimicrobial stewardship policies on COVID-19 wards were highlighted.

**Supplementary Information:**

The online version contains supplementary material available at 10.1186/s12941-023-00575-1.

## Introduction

The COVID-19 pandemic has impacted almost all areas of public health and we are only now beginning to see the full consequences. Whilst 98% of African countries have published data on COVID-19, there are limited reports on the impact of COVID-19 on hospital acquired infections (HAIs) and antimicrobial resistance (AMR) [1, 2]. Low- and middle-income countries (LMICs) are projected to be potential hot spots for AMR and the morbidity and mortality associated with it, and there are multiple factors driving this association, such as high numbers of informal urban settlements and the sanitation issues that accompany them, and a lack of public awareness of AMR [3, 4]. Widespread informal use of medications in many LMICs may complicate the situation further as unregulated access and a lack of clinical involvement in choice and duration may lead to inappropriate treatment of non-bacterial infections, as seen in viral diseases such as COVID-19 and influenza [5, 6]. Furthermore, the impact of COVID-19 lockdown measures and pressures on healthcare systems mean patients may have had reduced access to medical care and relied more heavily on unregulated sources of medical advice and antibiotics [7]. Preparedness for AMR is low in almost all countries across the African continent and the added pressure of a pandemic situation is likely to further exacerbate the situation [8].

Patients who develop severe or critical COVID-19 illness, defined as those requiring oxygen support, are more likely to acquire co-infections, such as bacterial pneumonia, that can be difficult to differentiate from COVID-19, and the WHO recommends empiric antibiotics to treat all likely pathogens [9, 10]. In mild to moderate cases of COVID-19, the use of antibiotics is discouraged, unless there is clinical suspicion of bacterial infection, to reduce the risk of the short-term side-effects of antibiotics for patients, as well as the potential long-term threats associated with increased AMR [11].

Despite the WHO’s guidance, the global use of antibiotics has been generally high, with reports suggesting between 50 and 95% of hospitalised COVID-19 patients receive antimicrobials [12–17]. At the beginning of the pandemic, bacterial HAIs have been reported in between 1 and 15% of COVID-19 patients, although far larger percentages have been observed [12–16, 18], and in approximately half of those who died from COVID-19 infection in China [13]. A study in Israel reported that having COVID-19 as well as a secondary bacterial infection increased a patient’s risk of death 2.7-fold [19]. In many countries where COVID-19 isolation wards have been set up, there have been changes in antimicrobial stewardship (AMS). In the UK for example, the use of broad-spectrum antimicrobials to treat secondary bacterial infections associated with prolonged intensive care admissions increased [14].

Changes in other infection prevention and control (IPC) practices in outbreak situations, e.g. COVID-19 wards, such as the increased use of personal protective equipment (PPE) and hand hygiene, may play a role in HAI prevalence, cause and resistance patterns. Outbreaks of resistant bacterial infections, including Methicillin-resistant *Staphylococcus aureus* (MRSA), have been reported in hospitals during outbreaks of novel emerging pathogens such as SARS in Hong Kong [20, 21]. When the WHO audited IPC practices, the African region’s mean score was the lowest of any region and no healthcare facilities in low-income countries met the IPC assessment framework requirements [22]. Rates of COVID-19 have varied across Africa, and countries have implemented differing responses, from the closures of schools and businesses, but not curfews or lockdowns (e.g. Tanzania and Zambia) to periods of country-wide home confinement (e.g. Sudan and Zimbabwe) [23].

The impact of contracting severe COVID-19, requiring hospitalisation, on a patient’s risk of developing a secondary bacterial infection, and the likelihood of resistance has not been widely studied in resource limited settings. Whilst there are a number of editorials and opinion pieces, there are few data from the African continent [11]. It is critical to reduce the risk of seriously ill COVID-19 patients contracting HAIs, whilst also trying to protect healthcare workers from COVID-19, and a greater understanding of the links between severe COVID-19 infection, HAIs, AMR and COVID-19 ward IPC and AMS can help to ensure the safety of both patients and staff. The primary aim of this study was to ascertain whether being admitted to a COVID-19 ward (with COVID-19 infection) compared to a non-COVID-19 ward (as a COVID-19 negative patient) was associated with a change in the prevalence of antimicrobial drug resistance. The secondary aims were to identify whether the distribution of species in HAIs was affected, and whether there were differences in AMS and IPC guidelines between COVID-19 and non-COVID-19 wards. The study was conducted in Sudan and Zambia, two resource constrained settings with differing country-wide responses to COVID-19.

## Methods

### Study design and setting

A cross sectional, hospital-based study was conducted in two countries, Sudan and Zambia, chosen for their differing COVID-19 response procedures [23]. Wards treating patients diagnosed with (and testing positive for) COVID-19 and comparator wards, treating patients who had not been diagnosed with COVID-19, were compared.

### Study population and sample size

Patients were enrolled until the specified sample size was reached for each ward in each country. All inpatients on COVID-19 wards (with a positive COVID-19 diagnosis) and on non-COVID-19 wards (patients who were COVID-19 negative when tested upon admission), with clinical evidence, based upon the guidelines for each hospital, of a bacterial HAI were eligible to be recruited to the study. HAIs were defined as infections that developed at least 48 h (Zambia) [24] and 72 h (Sudan) after admission, as per country guidelines [25, 26]. At the time of this study, no data were available for African countries, so assuming a mean secondary bacterial infection prevalence of around 25% on non-COVID-19 wards and a prevalence of 50% on COVID-19 wards across the study period (based on the literature at the beginning of the COVID-19 pandemic) [13, 15, 27, 28], a sample size estimate of 50 non-COVID-19 patients and 50 COVID-19 patients at each site, totalling 100 patients per site, and 200 overall was calculated based on 95% confidence level and a statistical power of 80% [29, 30].

In Sudan, data were collected between February-September 2021, which coincided with fluctuating case numbers of COVID-19 [31]. Patients were recruited from Soba University Hospital, Khartoum and private hospitals, with general surgery wards used as non-COVID-19 comparator wards. Data were collected between June and October 2021 from the University Teaching Hospital in Lusaka, Zambia, which coincided with the country’s third wave of COVID-19 [32]. To create a COVID-19 ward, a general surgery ward had been split, providing a non-COVID-19 comparator ward.

### Data collection

Length of ward stay and antibiotic prescribing data were collected for all patients recruited to the study. HAI prevalence data was also collected for all patients, not just those recruited to this study, admitted to the participating wards for the duration of the study period. The number of patients with resistant HAIs during the study period was collected for Sudan but was not available from Zambia. All data were analysed using Prism v9.4.1 (GraphPad).

### Isolate collection and microbiological testing

Isolates were collected as standard of care and further characterised using microbiological and biochemical methods, including API E and API NE panels (bioMérieux®) [33] and phenotypic antibiotic susceptibility testing (AST) using disc diffusion (Oxoid™) [34], following local standard operating procedures and based on performance standards for AST guidelines from the CLSI [35, 36].

### DNA extraction

A subset of isolates for whole genome sequencing (WGS) were selected based on their species identification and resistance phenotype. Isolates with phenotypic multi-drug resistant (MDR) profiles (defined as being resistant to at least one antimicrobial in three or more classes) were categorised as high priority, non-MDR *K. pneumoniae*, *E. coli* or MRSA were classed as medium priority and any other isolate as low priority. In Sudan, DNA from 24 isolates was extracted using the G-Spin™ Total DNA Extraction Kit (iNtRON Biotechnology) following manufacturer instructions (protocol F bacteria). In Zambia, DNA from 11 isolates was extracted using Qiagen DNA Mini Kit (following manufacturer’s instructions). Extracted DNA quality was evaluated using Qubit™ dsDNA BR Assay Kit (Thermo Fisher) and by agarose gel electrophoresis [37].

### Whole genome sequencing

A DNA library was prepared using the ONT Rapid Barcoding Kit (SQK-RBK004), following manufacturer’s instructions, and using 400 ng DNA per extracted isolate [38]. Up to twelve barcoded isolates were run on an R9.4.1 flow cell (ONT) using the Mk1C device for 48 h, using the default parameters on the Mk1C MinKNOW (v21.11.7) software. Basecalling was performed using Guppy (v6.0.6) and the flip-flop fast algorithm.

### Sequencing data analysis

Sequencing data was quality checked using FastQC (version 0.11.9) [39] and MultiQC (version 1.10.1) [40]. Fastq files were mapped to a reference genome (see supplementary data, table S4) using MiniMap2 (v2.20) [41] and polished using Medaka (v1.3.4) [42]. Assembled files were uploaded to the Centre of Genomic Epidemiology for analysis using KmerResistance 2.2 [43, 44], PlasmidFinder 2.1 [45, 46] and MLST 2.0 [46–52] to identify resistance genes, plasmids and types. The sequences were deposited in GenBank (supplementary data, Tables S5 and S6).

### IPC data collection

All versions of IPC guidelines for both COVID-19 and non-COVID-19 procedures that were in use during the study period were obtained with permission from the Sudanese and Zambian Ministries of Health, and specific guidelines for the participating hospitals were obtained [53–59]. The documents were summarised for core IPC measures and specific measures for COVID-19 management and antibiotic use (see supplementary data, table S1). Categories of IPC measures were compared between COVID-19 and general (pre-COVID-19) IPC guidelines. Any documents not published in English were translated and back translated to ensure accuracy.

## Results

### Rate of HAIs and bacterial prevalence

At the ward level, there were significantly more patients who developed HAIs (either susceptible or resistant) on COVID-19 wards (26%) compared to non-COVID-19 wards (6%) in Sudan (*p* = < 0.0001), although there was no significant difference (SD) in the number of patients with resistant HAIs (see Table 1). For Zambia, significantly more HAIs (both susceptible and resistant) (*p* = < 0.0001) occurred on the non-COVID-19 ward (21%) compared to COVID-19 wards (11%). The data for total number of patients with resistant HAIs was not available for Zambia.

**Table 1:** Descriptive summary of HAIs on non-COVID-19 and COVID-19 wards in Sudan and Zambia. S = susceptible, R = resistant. There were significantly more HAIs (both susceptible and resistant) isolated on COVID-19 wards compared to non-COVID-19 wards in Sudan, whilst the opposite was found in Zambia. There was no significant difference in the number of patients with resistant HAIs between the non-COVID-19 and COVID-19 wards in Sudan. These data were unavailable in Zambia

	Sudan		Zambia	
	Non-COVID-19 ward n (%)	COVID-19 ward n (%)	Non-COVID-19 ward n (%)	COVID-19 ward n (%)
Study period	Feb – Sept 2021	Feb-Oct 2021	Jun – Oct 2021	Jun-Sept 2021
Total patients during study period	3,959	858	317	514
No. patients with HAI (both S and R)	230 (6%)	208 (24%)	67 (21%)	54 (11%)
Significance	**** *p* = ≤ 0.0001		**** *p* = ≤ 0.0001	
No. patients with R HAI	203 (88%)	172 (83%)	Data not available	Data not available
Significance	Not significant (*p* = 0.1035)		n/a	

At study level, there was no significant difference in the number of antibiotic resistant isolates per patient on COVID-19 wards compared with non-COVID-19 wards in either Sudan or Zambia (Table 2). There was no significant difference in the length of time patients stayed on the wards in Sudan, but in Zambia, non-COVID-19 patients spent significantly more (*p* = 0.0182) time on the ward (mean of 38 days) compared to COVID-19 patients (mean of 30 days) (Table 2)

**Table 2:** Study level data. There was no SD in the mean number of resistant isolates per patient between the non-COVID-19 and COVID-19 wards in either country (resistances based on phenotypic data). There was no SD in length of ward stay in Sudan, but non-COVID-19 patients spent significantly longer on wards than COVID-19 patients in Zambia. Fisher’s exact test was applied in all cases

	Sudan		Zambia	
	Non-COVID-19 ward n (%)	COVID-19 ward n (%)	Non-COVID-19 ward n (%)	COVID-19 ward n (%)
Mean no. resistant isolates per patient	1.1	1.0	1.6	1.5
Significance	Not significant		Not significant	
Mean length of ward stay (days) (range)	11 (5–30)	15 (6–33)	38 (12–97)	30 (8–62)
Significance	Not significant		* *p* = 0.0182	

**Fig. 1:**
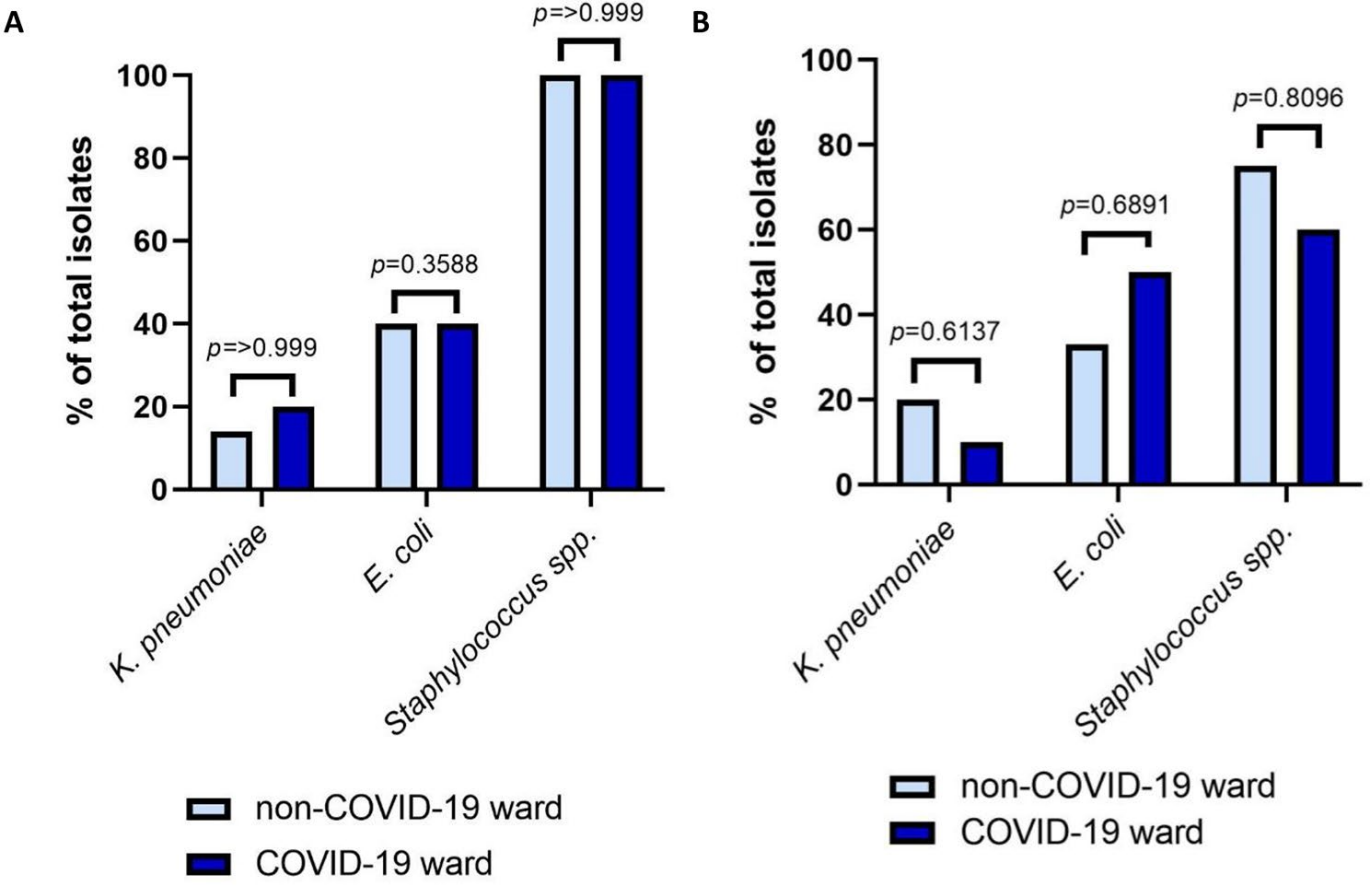
No SD in prevalence was found in the three most commonly seen species, *K. pneumoniae*, *E. coli* or *Staphylococcus* spp. between patients on the non-COVID-19 ward and COVID-19 wards in either (A) Sudan or (B) Zambia. Fisher’s exact test was applied in all cases. Note that the percentages for *K. pneumoniae* and *E. coli* relate to total numbers of Gram-negative isolates, and the percentage for *Staphylococcus* spp. is for total numbers of Gram-positive isolates

The number and prevalence of each species isolated using culture methods from patients on the COVID-19 and non-COVID-19 wards were compared in both countries. *E. coli* (n = 27 Sudan, n = 10 Zambia) and *K. pneumoniae* (n = 6 Sudan, n = 4 Zambia) were the two most commonly isolated gram-negatives and *Staphylococcus* spp. (n = 25 Sudan, n = 27 Zambia) the most common gram-positive, from patients across both types of ward in both countries. No significant difference was found in the prevalence of any species isolated from patients on COVID-19 wards compared to non-COVID-19 wards using Fisher’s exact test (Fig. 1). A list of Gram-positive and Gram-negative species identified on each ward can be found in the supplementary data (Tables S2 and S3).

### Phenotypic resistance

**Fig. 2:**
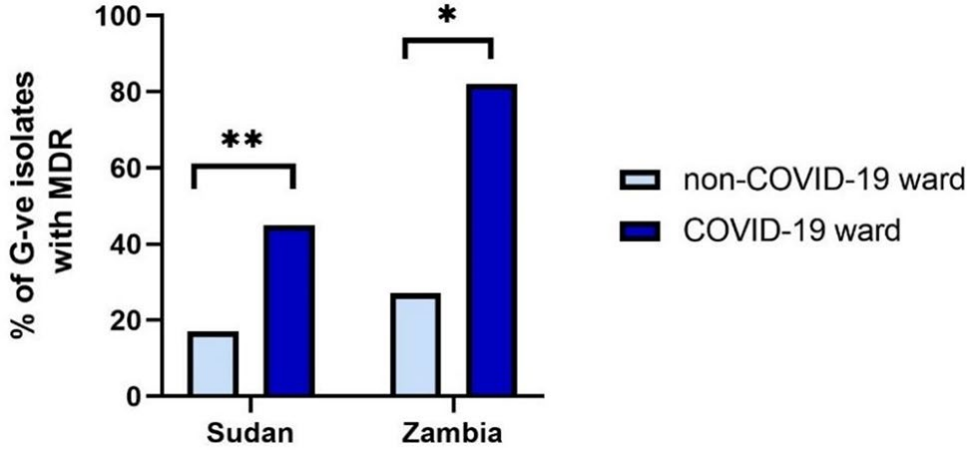
Percentage of isolates from Sudan and Zambia with MDR. There was a SD in the number of MDR isolates on non-COVID-19 compared to COVID-19 wards in both countries, *p* = 0.0087 in Sudan and *p* = 0.0154 in Zambia

In both countries there was a significant increase on COVID-19 wards in the number of phenotypic MDR Gram-negative isolates using Fisher’s exact test (*p* = 0.0087 in Sudan and *p* = 0.0154 in Zambia) (Fig. 2). There was no significant difference in the proportion of isolates with MRSA, Extended Spectrum Beta Lactamase (ESBL), Vancomycin-resistant enterococci (VRE) or Carbapenem-resistant Enterobacterales (CRE) resistances between COVID-19 and non-COVID-19 wards in either country. There were high levels of β-lactam resistance seen in isolates from both countries; in Sudan 95% (non-COVID-19) and 98% (COVID-19) and in Zambia 80% (non-COVID-19) and 100% (COVID-19). A list of phenotypic resistances and the number of isolates identified as resistant to each antibiotic tested can be found in the Supplementary data (Table S7).

**Fig. 3:**
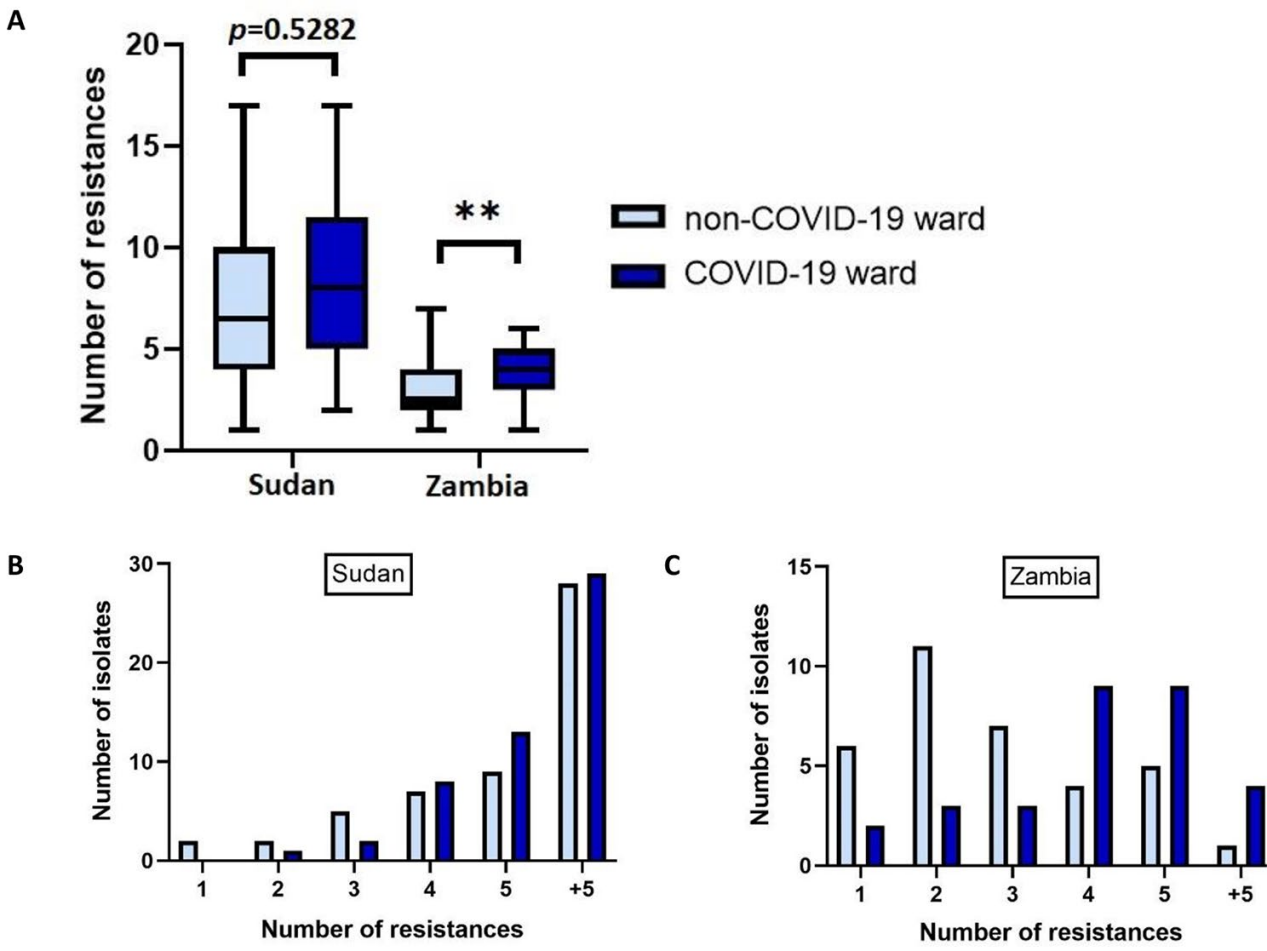
(A) box and whisker chart to show the mean number of phenotypic resistances per isolate from patients on non-COVID-19 and COVID-19 wards in Sudan and Zambia. There was no SD between patients on the wards in Sudan, but isolates had significantly more resistances from patients on the COVID-19 ward compared to the non-COVID-19 ward (*p* = 0.0036) in Zambia. The number of isolates with differing numbers of resistances were stratified, showing (B) the increasing number of isolates with greater numbers of phenotypic resistances from patients on both non-COVID-19 and COVID-19 wards in Sudan and (C) the increasing number of isolates with greater numbers of phenotypic resistances from patients on COVID-19 wards, but not on non-COVID-19 wards, in Zambia

In Sudan, no significant difference was found between patients on non-COVID-19 (mean = 6) and COVID-19 (mean = 8) (standard deviation = ± 4.56) wards when the number of phenotypic resistances per isolate for Gram-negatives was compared using Fisher’s exact test. When phenotypic resistances were categorised, there was no significant difference between any class (using Fisher’s exact test). In Zambia however, there was a significant difference (mean = 3 non-COVID-19, mean = 4 COVID-19, standard deviation = ± 1.66), using Fisher’s exact test (Fig. 3A, *p* = 0.036). Stratifying by number of resistances per isolate more clearly shows the correlation in both wards in Sudan (Fig. 3B).

In patients on the COVID-19 ward in Zambia the correlation between number of isolates and phenotypic number of resistances was generally positive, but the relationship on the non-COVID-19 ward showed the opposite (Fig. 3C). There was a significant decrease in the total number of phenotypic resistances detected in Gram-negative isolates from patients on the non-COVID-19 ward (mean = 45, range = 30–79) and COVID-19 ward (mean = 19, range = 7–34) (*p* = 0.0036 using an independent t-test) and when phenotypic resistances were categorised, there was no SD between any antibiotic class.

### Genotypic resistance

**Fig. 4:**
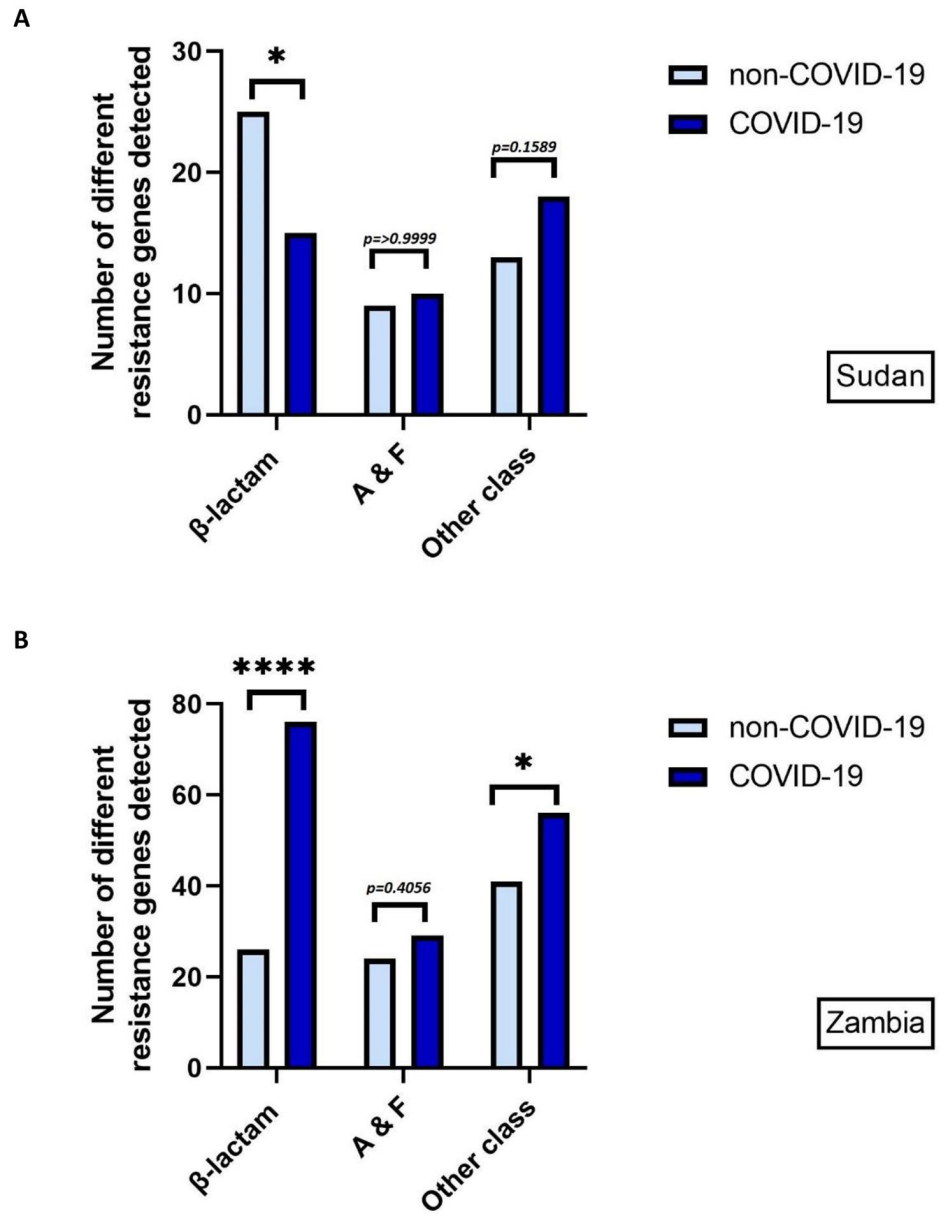
Total number of different β-lactamase, aminoglycoside and fluoroquinolone, and other antibiotic class resistance genes identified in patients from COVID-19 and non-COVID-19 wards in (A) Sudan and (B) Zambia isolate sequencing data. A = aminoglycosides, F = fluoroquinolones. NS = not significant

In Sudan, there was no significant difference (*p* = 0.5282) between the mean number of resistance genes detected in Gram-negative isolates from patients on the non-COVID-19 ward (mean = 15, range = 2–19) and COVID-19 ward (mean = 17, range = 5–26), using an independent t-test. When genes conferring resistance to different antibiotic classes were analysed, a significant difference in the number of β-lactamase genes (*p* = 0.0192) was identified, but no significant difference was found in genes conferring resistance to aminoglycosides and fluoroquinolones, or to other classes, using Fisher’s exact test (Fig. 4A).

In Zambia, when genes conferring resistance to different antibiotic classes were analysed, a significant increase the number of β-lactamase genes (*p* = ≤ 0.0001) and other classes (*p* = 0.0348) was identified, but no SD was found in genes conferring resistance to aminoglycosides and fluoroquinolones, using Fisher’s exact test (Fig. 4B).

Eleven β lactamase genes, 7 aminoglycoside and fluoroquinolone resistance genes and 17 other antibiotic class resistance genes were identified in Gram-negatives in this study (supplementary data, Table S8). Some genes, such as *aac*, were commonly found in both countries and in patients across both wards. In Sudan, 4 and 6 plasmids were identified in isolates from patients on non-COVID-19 and COVID-19 wards respectively. In Zambia 14 and 1 plasmid(s) were identified in an isolate from a patient on the non-COVID-19 and COVID-19 wards respectively (supplementary data, Table S9).

### Antibiotic prescribing and length of stay

Eligible patients who were diagnosed as COVID-19 positive were given antibiotics on admission in accordance with institutional and national guidelines or at the discretion of the attending physician. In most cases this was azithromycin, the first line recommended antibiotic for moderate diseases in both countries [55, 57]. Antibiotic protocols for HAIs were not reviewed. For the treatment of HAIs in Sudan, most COVID-19 patients were prescribed azithromycin and gentamicin (n = 36), with other combinations of a macrolide and an aminoglycoside also used. For non-COVID-19 patients, the cephalosporins ceftazidime or ceftriaxone (n = 40), often in combination with macrolides or metronidazole, were most commonly used (Table 3). Whilst azithromycin was not prescribed prophylactically or used to treat HAIs on the non-COVID-19 ward, 46% of HAIs isolated from that ward were resistant to it.

**Table 3:** Commonly prescribed antibiotics on non-COVID-19 and COVID-19 wards in Sudan, the number of patients with isolates resistant to them and how often they were prescribed as monotherapies. Resistance data taken from phenotypic ASTs

Non-COVID-19 ward				COVID-19 ward			
Antibiotic	Number of patients treated	Total no. patients with resistant isolates	No. times used as a monotherapy	Antibiotic	Number of patients treated	Total no. patients with resistant isolates	No times used as a monotherapy
Ceftazidime	35 (67%)	29 (56%)	6 (17%)	Gentamicin	39 (74%)	23 (43%)	0 (0%)
Vancomycin	19 (37%)	0 (0%)	0 (0%)	Azithromycin	39 (74%)	21 (40%)	2 (10%)
Ceftriaxone	14 (27%)	24 (46%)	0 (0%)	Ceftazidime	12 (23%)	37 (70%)	2 (17%)
Metronidazole	13 (25%)	Not reported	0 (0%)	Erythromycin	7 13%)	9 (17%)	0 (0%)
Ciprofloxacin	4 (8%)	30 (58%)	3 (75%)	Meropenem	3 (6%)	14 (26%)	0 (0%)

For Zambia, most patients were prescribed ceftriaxone for HAIs (n = 26 COVID-19, n = 18 non-COVID-19). Cotrimoxazole was the only antibiotic prescribed in combination (Table 4). Resistance to the most commonly prescribed antibiotic, ceftriaxone, was only noted in one patient. None of the isolates tested on either the COVID-19 or the non-COVID-19 ward showed resistance to azithromycin, which was used to treat one patient HAI on the COVID-19 ward and not at all for HAIs on the non-COVID-19 ward.

**Table 4:** Most commonly prescribed antibiotics on non-COVID-19 and COVID-19 wards in Zambia, the number of patients with isolates resistant to them and how often they were prescribed as monotherapies. Resistance data taken from phenotypic ASTs

Non-COVID-19 ward				COVID-19 ward			
Antibiotic	Number of patients treated	Total no. patients with resistant isolates	No. times used as a monotherapy	Antibiotic	Number of patients treated	Total no. patients with resistant isolates	No. times used as a monotherapy
Ceftriaxone	18 (38%)	1 (2%)	18 (100%)	Ceftriaxone	26 (52%)	0 (0%)	26 (100%)
Metronidazole	6 (13%)	Not reported	6 (100%)	Cloxacillin	3 (6%)	1 (2%)	3 (100%)
Cloxacillin	5 (10%)	0 (0%)	5 (100%)	Gentamicin	2 (4%)	11 (22%)	2 (100%)
Gentamicin	4 (8%)	7 (15%)	4 (100%)	Cotrimoxazole	2 (4%)	7 (14%)	2 (100%)
Cotrimoxazole	2 (4%)	4 (8%)	2 (100%)	Levofloxacin	2 (4%)	0 (0%)	2 (100%)

### Review of IPC guidelines

In both countries comprehensive IPC guidance existed prior to COVID-19 and covered all key elements including case definitions, roles and responsibilities, identification and isolation of infectious cases, hand hygiene and PPE, occupational health measures, decontamination and sterilisation of equipment, waste management, and hospital cleaning (supplementary data, Table S1). Guidance issued in response to COVID-19 emphasised criteria for disease severity and clinical management in both countries, including AMS guidance. Specific hospital documentation showed a variation in approach to IPC measures, with a practical checklist covering all operational aspects in Zambia and an operational protocol for Sudan, emphasising establishment of responsibility for different aspects of IPC management. Both sets reflected national guidance.

## Discussion

The significant increase in MDR seen in isolates from patients on COVID-19 wards in both Sudan and Zambia is concerning. The changing emphasis on IPC measures and prescribing practices, such as the prescription of antibiotics upon admission and the likelihood of being treated with antimicrobials for suspected pulmonary infection prior to diagnosis of COVID-19 may be driving a change in HAIs and AMR patterns on COVID-19 wards. However, the picture is complex, with patient factors such as comorbidities, disease severity and reason for original admission potentially having an effect.

The increase in the number of phenotypic resistances per isolate seen in patients on COVID-19 wards (significantly so in Zambia) was not echoed by the mean number of resistance genes per isolate seen; in Sudan there was no difference when isolates from the two wards were compared, and in Zambia there was a significant decrease in mean number of resistance genes per isolate from patients on the COVID-19 ward, indicating that the AMR picture is just as complicated. Whilst the introduction of COVID-19 specific IPC measures may not have altered acquisition of specific genotypic resistance mechanisms, it may have increased the prevalence of non-specific mechanisms, such as efflux pumps, affecting multiple antibiotics. An increase in phenotypic resistance, but not in genotypic resistance markers may also indicate changes in regulatory-based mechanisms rather than genetic control. The increase in MDR isolates found in patients on COVID-19 wards indicates an enhanced fitness of multiple-resistance phenotypes and a shift towards the selection for them in these settings.

The fact that no differences were seen in other resistance patterns, including MRSA, ESBL, VRE or CRE, and that there were no changes in the proportion of the species isolated is a positive sign that being admitted to a COVID-19 ward may be having a limited, albeit important, effect on the resistances seen in HAIs in COVID-19-positive patients. Identifying the cause, whether due to changes in IPC and AMS, patient factors, something else, or a combination of issues, is likely to be complicated. Whilst IPC measures were not explicitly changed for COVID-19 wards in either country, guidance in both emphasised the adherence to and continued use of existing IPC guidance. IPC changes and compliance are difficult to quantify using guidelines alone and it was not possible to establish a clear causal relationship in this study [60, 61]. Systematically measuring the use of PPE, hand hygiene and ward cleaning routines and its effect on AMR and HAI transmission merits further research [62, 63]. All study sites were large, tertiary hospitals, so identifying whether these guidelines were changed to a greater or lesser degree in response to COVID-19 in other healthcare levels would also be of interest.

In this study, all patients admitted to COVID-19 wards in both countries were automatically prescribed antibiotics, regardless of disease severity. It was noted that clinical prescribing would also be influenced by other factors such as allergies, other medications, nature of secondary infection, patient factors and antibiotic availability locally, as well as clinician experience. Clinical evaluation of patients followed the guidelines for each hospital and country. Whilst bacterial infection guidelines were not evaluated as part of this study, differences in clinical evaluation and prescribing should be factored into potential differences between the countries. Whilst the comprehensive approach of treating all hospitalised COVID-19 patients with empiric antibiotics did follow country guidance, it does not comply with the WHO’s COVID-19 clinical management policy [9]. Azithromycin is commonly recommended for early treatment of COVID-19 and mild to moderate respiratory tract infections due to its safety record and efficacy against common respiratory pathogens and is freely available in the community in both Sudan and Zambia [64, 65]. This common community use, pre-COVID-19, could be a factor in the resistance patterns seen, particularly in Sudan [66]. This will be difficult to track, as there are currently few data available on global rates of azithromycin resistance, although some studies suggest currently low, but increasing levels of resistance [67, 68].

Whilst less resistance to commonly used antibiotics were reported in Zambia, that metronidazole is not captured in the AST guidelines requires consideration, as it being commonly prescribed in both settings for HAIs. This emphasises the need for up-to-date AMR surveillance, providing evidence for a review of AST panels, to include the most current and frequently used antibiotics globally. The use of antibiotics in the early stages of illness prior to hospital admission, and those prescribed upon admission to hospital, are likely to affect the resistances seen in HAIs. This merits a review of wider antibiotic guidance with a view to balancing efficacy against the risk of developing long-term resistance. There is also a potential opportunity for stronger focus on IPC measures to reduce HAI and thus reduce the possibility for further resistance and transmission. Compared to the previous literature, the prevalence of HAIs on both COVID-19 and non-COVID-19 wards found in this study were lower. However, there are few data for this from African countries, and so further studies across the continent would help elucidate this.

In Sudan, the levels of resistance found in isolates from patients on both non-COVID-19 and COVID-19 wards and the percentage of isolates resistant to multiple first line antibiotics suggests IPC practices that may predate the COVID-19 pandemic. Whilst antibiotic prescribing was found to be different for patients diagnosed with COVID-19 compared to those on the non-COVID-19 comparator wards, apart from increases in the overall number of HAIs and the number of MDR isolates, it did not affect other AMR patterns seen on the wards. This particularly applied when the genotypic data were examined. The significantly higher levels of β-lactamase genes seen in isolates from patients on the non-COVID-19 ward should be noted, considering that β-lactams were the most commonly prescribed antibiotics on that ward.

In Zambia, the longer mean length of ward stay for non-COVID-19 patients may be a factor in why non-COVID-19 patients acquired more HAIs. This is because non-severe COVID-19 patients were more likely to be sent home due to a lack of bed availability, as this study was conducted at the peak of the third COVID-19 wave in Zambia [32]. The decreased number of total HAIs seen in patients on the COVID-19 ward suggests that enhanced IPC measures may have also reduced levels of HAI transmission. Despite the overall decrease in HAIs seen in patients on the COVID-19 ward, those isolates that were resistant showed higher numbers of resistances, both phenotypically and genotypically. The fact that antibiotic prescribing was similar on both the COVID-19 and non-COVID-19 ward suggests that other IPC measures may play a more significant role.

This study was set up under the auspices of a BSAC award and training in sequencing of AMR isolates was a key output. This aspect of the study encountered complications related to COVID-19 restrictions, as travel constraints meant that all training was conducted online, but all WGS was performed in Sudan and Zambia. Originally intended specifically for the sites within the study, training materials have since been published enabling others to learn from our experiences [69].

## Conclusions

The data from this study presents a complex picture of AMR and an increase in cases of MDR in HAIs in patients who are COVID-19 positive, suggesting the possibility of an AMR pandemic within the COVID-19 pandemic (see Table 5). The changes in HAIs and AMR patterns seen in COVID-19 patients on COVID-19 wards compared to COVID-19 negative patients on non-COVID-19 wards in Sudan and Zambia are likely due to a potentially complex combination of causes, including patient factors such as original reason for hospitalisation, comorbidities and severity of illness, as well as differing emphases on infection prevention and control, and antimicrobial stewardship policies on COVID-19 wards. Identifying the impact of IPC responses and how to balance the protection of patients and staff, as well as limiting HAIs and AMR in pandemic situations would be both practically and financially astute [70]. Further studies to help understand the links in this multifaceted picture are vital to protect patients and healthcare workers from both COVID-19 infection and HAIs, and protecting antibiotics from increasing multi-drug resistant pathogens.

**Table 5:** Summary of findings from this study

COVID-19 wards compared with non-COVID-19 wards:	Sudan	Zambia
**Patient data**		
Antibiotic prescribing	Different	Similar
Length of ward stay	No difference	Decreased (SD)
**Phenotypic data**		
Species prevalence	No difference	No difference
Number of HAIs (susceptible and resistant)	Increased (SD)	Decreased (SD)
Number of resistant HAIs	No difference	No data available
Number of MDR isolates	Increased (SD)	Increased (SD)
Number of resistant isolates per patient	No difference	No difference
Number of resistances per isolate	Increased	Increased (SD)
**Genotypic data**		
Number of β-lactam resistant isolates	Decreased (SD)	Increased (SD)
Number of aminoglycoside and fluoroquinolone resistant isolates	No difference	No difference
Number of isolates resistant to other antibiotic classes	No difference	Increased (SD)
Total number of different plasmids identified	No difference	Decreased
Number of resistance genes per isolate	No difference	Decreased (SD)

## Electronic supplementary material

Below is the link to the electronic supplementary material.

Supplementary Table S1: Table comparing key IPC documentation for each setting

Supplementary Table S2: List of gram-negative isolates

Supplementary Table S3: List of gram-positive isolates

Supplementary Table S4: Accession numbers of reference genomes used in this study

Supplementary Table S5: Accession numbers of isolates collected in Sudan

Supplementary Table S6: Accession numbers of isolates collected in Zambia

Supplementary Table S7: Number of isolates from each ward showing phenotypic resistances to each antibiotic tested

Supplementary Table S8: Antibiotic resistance genes identified in Gram-negatives this study. The heat map shows the percentage of isolates sequenced on each ward that had at least one version of the gene present

Supplementary Table S9: Number of plasmids found on each ward and the percentage of isolates on each ward that they were identified in

## Data Availability

The datasets supporting the conclusions of this article are included within the article and its additional files. Sequencing data is available under BioProject PRJNA806525 (for Sudan) and PRJNA880679 (for Zambia).

## List of abbreviations

AMR: Antimicrobial resistance
AMS: Antimicrobial stewardship
AST: Antibiotic sensitivity test
CRE: Carbapenem resistant Enterobacterales
ESBL: Extended Spectrum Beta Lactamase
HAI: Hospital acquired infection
HIC: High income country
IPC: Infection prevention and control
LM: Lower-middle (income country)
LMICs: Low- and middle-income countries
MDR: Multi-drug resistant
MRSA: Methicillin-resistant *Staphylococcus aureus*
NS: Not significant
ONT: Oxford Nanopore Technologies
PPE: Personal protective equipment
SD: Significant difference
VRE: Vancomycin-resistant enterococci
WGS: Whole genome sequencing
WHO: World Health Organization
