# Supplementary material for: A pandemic within a pandemic? Admission to COVID-19 wards in hospitals is associated with increased prevalence of antimicrobial resistance in two African settings
Source: Ann Clin Microbiol Antimicrob. 2023 Apr 13;22:25. doi: 10.1186/s12941-023-00575-1 (PMC10101537; doi:10.1186/s12941-023-00575-1)
Supplement: Supplementary file 1 — Supplementary Table S1: Table comparing key IPC documentation for each setting [file 12941_2023_575_MOESM1_ESM.docx]

|  | **Zambia** | | | **Sudan** | | |
| --- | --- | --- | --- | --- | --- | --- |
|  | **Infection Prevention and Control Water Sanitation and Hygiene in Health Care Facilities: The Guidelines and Standard Operation Procedures** [23] | **Interim Clinical Guidance for Management of Patients with Coronavirus Disease 2019** [24] | **Health Facility Infection Prevention and Control Monitoring Tool, University Teaching Hospital, Zambia** [27] | **National Infection prevention and Control Manual** [22] | **Case Treatment Protocol For COVID-19 Patients** [25] | **Soba University Hospital Manual Operation Guideline** [28] |
| General summary | 66-page document addressing IPC and water, sanitation and hygiene | 41-page document addressing IPC and clinical management of COVID-19 patients | 17-page checklist of IPC implementation measures | 268-page document addressing all aspects of IPC | 9-page treatment protocol for COVID-19 patients | 60-page operational document addressing IPC measures in Soba University Hospital |
| Summary of contents | Specific mention of   - Water and sanitation standards - Hand hygiene - Decontamination, sterilisation, and cleaning - Equipment and infrastructure - PPE   Includes content for monitoring IPC measures | Specific mention of   - Triage approach, including sample collection - Use of PPE - Clinical management of patients, including those treated at home - IPC measures including hand hygiene, PPE, distancing, ventilation - Management of contacts of cases - Cleaning, sterilisation, and waste management | Checklist reviewing IPC management, guidelines and SOPs, including:   - Education materials for patients - PPE availability and use - Isolation - Hand hygiene - Occupational health - Water and sanitation - Cleaning sterilisation and waste management | General prevention measures including   - Hand hygiene - Isolation - PPE - Surgical IPC measures - Cleaning, sterilisation, and waste management - Surveillance and outbreak management - Occupational health - IPC measures in specific hospital settings e.g. ICU, laboratory | IPC guidance advises:   - Follow standard IPC guidance - Use of PPE - Specific areas to assess COVID patients | IPC guidance covering   - Hand hygiene - PPE - Occupational health - Surgical IPC measures - Cleaning, sterilisation, and waste management - Isolation - IPC measures in specific hospital settings eg ICU |
| COVID-19 management approach | 3 levels of disease severity with treatment approach for each, including guidance for cases treated outside of hospital  Specific guidance for children, pregnant women and people living with HIV (PLHIV) | | | 4 levels of disease severity with treatment approach for each  Specific guidance or children and pregnant women | | |
| Antibiotics for COVID patients^[[1]](#footnote-1)^ | Non severe pneumonia   - Amoxicillin (oral)   Severe pneumonia   - CAP - Beta-lactam plus macrolide   HAP^[[2]](#footnote-2)^ – consider MDR organisms including pseudomonas, with specific guidance on pseudomonas treatment  (Beta lactam plus aminoglycoside, carbapenems with antipseudomonal, fluoroquinolone with aminoglycoside)  Specific guidance for PLHIV, pregnant women and children | | | Moderate disease, treat as CAP^[[3]](#footnote-3)^   - Amoxicillin + Azithromycin OR - Doxycycline   Severe disease   - Ceftriaxone + Azithromycin   Critical / sepsis   - Piperacillin /Tazobactam or Meropenem + Levofloxacin   Guidance if concerns for MRSA: (Vancomycin, Teicoplanin or Linezolid) | | |

Table S1. Table comparing key IPC documentation for each setting, pre and post COVID-19 at both national and local level

1. Note that antibiotic guidelines for non-COVID patients were not reviewed. [↑](#footnote-ref-1)
2. HAP – Hospital Acquired Pneumonia [↑](#footnote-ref-2)
3. CAP – Community Acquired Pneumonia [↑](#footnote-ref-3)
