# Supplementary material for: A pandemic within a pandemic? Admission to COVID-19 wards in hospitals is associated with increased prevalence of antimicrobial resistance in two African settings
Source: Ann Clin Microbiol Antimicrob. 2023 Apr 13;22:25. doi: 10.1186/s12941-023-00575-1 (PMC10101537; doi:10.1186/s12941-023-00575-1)
Supplement: Supplementary file 2 — Supplementary Table S2: List of gram-negative isolates [file 12941_2023_575_MOESM2_ESM.docx]

|  |  | |  | |
| --- | --- | --- | --- | --- |
|  |  |  |  |  |
|  |  |  |  |  |
|  |  |  |  |  |
|  |  |  |  |  |
|  |  |  |  |  |
|  |  |  |  |  |
|  |  |  |  |  |
|  |  |  |  |  |
|  |  |  |  |  |
|  |  |  |  |  |
|  |  |  |  |  |
|  |  |  |  |  |
|  |  |  |  |  |
|  |  |  |  |  |
|  |  |  |  |  |
|  |  |  |  |  |
|  |  |  |  |  |
|  |  |  |  |  |
|  |  |  |  |  |
|  |  |  |  |  |

|  | **Sudan** | | | | **Zambia** | | | |
| --- | --- | --- | --- | --- | --- | --- | --- | --- |
| **Species** | **Non-COVID-19 ward** | | **COVID-19 ward** | | **Non-COVID-19 ward** | | **COVID-19 ward** | |
|  | **n** | **Percentage** | **n** | **Percentage** | **n** | **Percentage** | **n** | **Percentage** |
| *Klebsiella pneumoniae* | 6 | 14% | 6 | 20% | 3 | 20% | 1 | 10% |
| *Escherichia coli* | 17 | 40% | 12 | 40% | 5 | 33% | 5 | 50% |
| *Pseudomonas spp.* | 7 | 17% | 3 | 10% | 0 | 0% | 0 | 0% |
| *Acinetobacter* | 2 | 5% | 1 | 3% | 0 | 0% | 0 | 0% |
| *Enterobacter cloacae* | 4 | 10% | 2 | 7% | 1 | 7% | 0 | 0% |
| Other *Gram-negative bacilli* | 1 | 2% | 1 | 3% | 0 | 0% | 0 | 0% |
| *Burkholderia cepacia* | 1 | 2% | 0 | 0% | 0 | 0% | 0 | 0% |
| *Pseudomonas luteola* | 3 | 7% | 1 | 3% | 0 | 0% | 0 | 0% |
| *Stenotrophomonas maltophilia* | 1 | 2% | 0 | 0% | 0 | 0% | 0 | 0% |
| *Aeromonas hydrophila* | 0 | 0% | 2 | 7% | 0 | 0% | 0 | 0% |
| *Klebsiella aerogenes* | 0 | 0% | 0 | 0% | 1 | 7% | 0 | 0% |
| *Proteus mirabilis* | 0 | 0% | 0 | 0% | 2 | 13% | 0 | 0% |
| *Acinetobacter baumannii* | 0 | 0% | 0 | 0% | 1 | 7% | 1 | 10% |
| *Enterobacter agglomerans* | 0 | 0% | 0 | 0% | 0 | 0% | 2 | 20% |
| *Pseudomonas aeruginosa* | 0 | 0% | 0 | 0% | 2 | 13% | 0 | 0% |
| *Enterobacter spp.* | 0 | 0% | 1 | 3% | 0 | 0% | 0 | 0% |
| *Serratia marcescens* | 0 | 0% | 1 | 3% | 0 | 0% | 0 | 0% |
| Total isolates | **42** |  | **30** |  | **15** |  | **10** |  |

Table S2. List of Gram-negative isolates isolated from non-COVID-19 and COVID-19 wards in both Sudan and Zambia.
