# Supplementary material for: A pandemic within a pandemic? Admission to COVID-19 wards in hospitals is associated with increased prevalence of antimicrobial resistance in two African settings
Source: Ann Clin Microbiol Antimicrob. 2023 Apr 13;22:25. doi: 10.1186/s12941-023-00575-1 (PMC10101537; doi:10.1186/s12941-023-00575-1)
Supplement: Supplementary file 3 — Supplementary Table S3: List of gram-positive isolates [file 12941_2023_575_MOESM3_ESM.docx]

|  |  | |  | |
| --- | --- | --- | --- | --- |
|  |  |  |  |  |
|  |  |  |  |  |
|  |  |  |  |  |
|  |  |  |  |  |
|  |  |  |  |  |

|  | **Sudan** | | | | **Zambia** | | | |
| --- | --- | --- | --- | --- | --- | --- | --- | --- |
| **Species** | **Non-COVID-19 ward** | | **COVID-19 ward** | | **Non-COVID-19 ward** | | **COVID-19 ward** | |
|  | **n** | **Percentage** | **n** | **Percentage** | **n** | **Percentage** | **n** | **Percentage** |
| *Staphylococcus spp.* | 12 | 100% | 13 | 100% | 19 | 100% | 15 | 83% |
| *Enterococcus faecium* | 0 | 0% | 0 | 0% | 0 | 0% | 1 | 6% |
| *Viridans group Streptococcus* | 0 | 0% | 0 | 0% | 0 | 0% | 2 | 11% |
| Total | **12** |  | **13** |  | **19** |  | **18** |  |

Table S3. List of Gram-positive isolates isolated from non-COVID-19 and COVID-19 wards in both Sudan and Zambia
