# Supplementary material for: A pandemic within a pandemic? Admission to COVID-19 wards in hospitals is associated with increased prevalence of antimicrobial resistance in two African settings
Source: Ann Clin Microbiol Antimicrob. 2023 Apr 13;22:25. doi: 10.1186/s12941-023-00575-1 (PMC10101537; doi:10.1186/s12941-023-00575-1)
Supplement: Supplementary file 4 — Supplementary Table S4: Accession numbers of reference genomes used in this study [file 12941_2023_575_MOESM4_ESM.docx]

| **Species** | **Genome** | **Accession number** |
| --- | --- | --- |
| *E. coli* | K12 | U00096.3 |
| *K. pneumoniae* | HS11286 | CP003200.1 |
| *Burkholderia cepacia* | AU41368 | NZ_JAIZPY000000000.1 |
| *Aeromonas hydrophila* | OnP3.1 | NZ_CP050851.1 |
| *Acinetobacter baumannii* | n/a | NZ_CP043953.1 |
| *Pantoea agglomerans* | n/a | NZ_CP077366.1 |
| *Proteus mirabilis* | HI4320 | NC_010554.1 |

Table S4. Accession numbers of reference genomes used in this study.
