# Supplementary material for: A pandemic within a pandemic? Admission to COVID-19 wards in hospitals is associated with increased prevalence of antimicrobial resistance in two African settings
Source: Ann Clin Microbiol Antimicrob. 2023 Apr 13;22:25. doi: 10.1186/s12941-023-00575-1 (PMC10101537; doi:10.1186/s12941-023-00575-1)
Supplement: Supplementary file 5 — Supplementary Table S5: Accession numbers of isolates collected in Sudan [file 12941_2023_575_MOESM5_ESM.docx]

| **Accession** | **Ward** | **Organism** | **BioProject** |
| --- | --- | --- | --- |
| SAMN26095812 | COVID-19 | *Klebsiella pneumoniae* | PRJNA806525 |
| SAMN26095813 | COVID-19 | *Klebsiella pneumoniae* | PRJNA806525 |
| SAMN26095814 | COVID-19 | *Klebsiella pneumoniae* | PRJNA806525 |
| SAMN26095815 | COVID-19 | *Klebsiella pneumoniae* | PRJNA806525 |
| SAMN26095816 | Non-COVID-19 | *Escherichia coli* | PRJNA806525 |
| SAMN26095817 | Non-COVID-19 | *Escherichia coli* | PRJNA806525 |
| SAMN26095818 | Non-COVID-19 | *Escherichia coli* | PRJNA806525 |
| SAMN26095819 | Non-COVID-19 | *Escherichia coli* | PRJNA806525 |
| SAMN26095820 | Non-COVID-19 | *Escherichia coli* | PRJNA806525 |
| SAMN26095821 | COVID-19 | *Escherichia coli* | PRJNA806525 |
| SAMN26095822 | COVID-19 | *Escherichia coli* | PRJNA806525 |
| SAMN26095823 | COVID-19 | *Escherichia coli* | PRJNA806525 |
| SAMN26095824 | COVID-19 | *Escherichia coli* | PRJNA806525 |
| SAMN26095825 | COVID-19 | *Escherichia coli* | PRJNA806525 |
| SAMN26095826 | Non-COVID-19 | *Escherichia coli* | PRJNA806525 |
| SAMN26095827 | COVID-19 | *Escherichia coli* | PRJNA806525 |
| SAMN26095828 | COVID-19 | *Escherichia coli* | PRJNA806525 |
| SAMN26095829 | Non-COVID-19 | *Escherichia coli* | PRJNA806525 |
| SAMN26095830 | Non-COVID-19 | *Escherichia coli* | PRJNA806525 |
| SAMN26095831 | Non-COVID-19 | *Escherichia coli* | PRJNA806525 |
| SAMN26095832 | Non-COVID-19 | *Burkholderia cepacia* | PRJNA806525 |
| SAMN26095833 | COVID-19 | *Aeromonas hydrophila* | PRJNA806525 |
| SAMN26095834 | COVID-19 | *Aeromonas hydrophila* | PRJNA806525 |
| SAMN26095835 | COVID-19 | *Escherichia coli* | PRJNA806525 |
| SAMN26096491 | COVID-19 | *Klebsiella pneumoniae* | PRJNA806525 |
| SAMN26096492 | COVID-19 | *Klebsiella pneumoniae* | PRJNA806525 |
| SAMN26096493 | Non-COVID-19 | *Klebsiella pneumoniae* | PRJNA806525 |
| SAMN26096494 | Non-COVID-19 | *Klebsiella pneumoniae* | PRJNA806525 |
| SAMN26096495 | COVID-19 | *Klebsiella pneumoniae* | PRJNA806525 |
| SAMN26096496 | COVID-19 | *Klebsiella pneumoniae* | PRJNA806525 |
| SAMN26096497 | Non-COVID-19 | *Klebsiella pneumoniae* | PRJNA806525 |
| SAMN26096498 | Non-COVID-19 | *Klebsiella pneumoniae* | PRJNA806525 |
| SAMN26096499 | Non-COVID-19 | *Klebsiella pneumoniae* | PRJNA806525 |
| SAMN26096500 | Non-COVID-19 | *Klebsiella pneumoniae* | PRJNA806525 |

Table S5. Accession numbers of isolates collected in Sudan.
