# Supplementary material for: A pandemic within a pandemic? Admission to COVID-19 wards in hospitals is associated with increased prevalence of antimicrobial resistance in two African settings
Source: Ann Clin Microbiol Antimicrob. 2023 Apr 13;22:25. doi: 10.1186/s12941-023-00575-1 (PMC10101537; doi:10.1186/s12941-023-00575-1)
Supplement: Supplementary file 6 — Supplementary Table S6: Accession numbers of isolates collected in Zambia [file 12941_2023_575_MOESM6_ESM.docx]

| **Accession** | **Ward** | **SPUID Organism** | **BioProject** |
| --- | --- | --- | --- |
| SAMN30356174 | COVID-19 | *Acinetobacter baumannii* | PRJNA880679 |
| SAMN30356175 | COVID-19 | *Viridans group Streptococcus* | PRJNA880679 |
| SAMN30356176 | COVID-19 | *Klebsiella pneumoniae* | PRJNA880679 |
| SAMN30356179 | COVID-19 | *Escherichia coli* | PRJNA880679 |
| SAMN30356178 | COVID-19 | *Pantoea agglomerans* | PRJNA880679 |
| SAMN30356177 | COVID-19 | *Escherichia coli* | PRJNA880679 |
| SAMN30356180 | Non-COVID-19 | *Proteus mirabilis* | PRJNA880679 |
| SAMN30356184 | Non-COVID-19 | *Klebsiella pneumoniae* | PRJNA880679 |
| SAMN30356183 | Non-COVID-19 | *Escherichia coli* | PRJNA880679 |
| SAMN30356182 | Non-COVID-19 | *Escherichia coli* | PRJNA880679 |
| SAMN30356181 | Non-COVID-19 | *Klebsiella pneumoniae* | PRJNA880679 |

Table S6. Accession numbers of isolates collected in Zambia.
