# Supplementary material for: A pandemic within a pandemic? Admission to COVID-19 wards in hospitals is associated with increased prevalence of antimicrobial resistance in two African settings
Source: Ann Clin Microbiol Antimicrob. 2023 Apr 13;22:25. doi: 10.1186/s12941-023-00575-1 (PMC10101537; doi:10.1186/s12941-023-00575-1)
Supplement: Supplementary file 7 — Supplementary Table S7: Number of isolates from each ward showing phenotypic resistances to each antibiotic tested [file 12941_2023_575_MOESM7_ESM.docx]

|  | **Sudan** | | **Zambia** | |
| --- | --- | --- | --- | --- |
|  | non-COVID-19 ward | COVID-19 ward | non-COVID-19 ward | COVID-19 ward |
| Amikacin | 18 | 15 | 0 | 0 |
| Amoxicillin | 0 | 0 | 0 | 1 |
| Amoxicillin + clavulanic acid | 24 | 19 | 2 | 0 |
| Ampicilin | 24 | 24 | 3 | 12 |
| Aztreonam | 24 | 21 | 0 | 0 |
| Cefazolin | 0 | 0 | 4 | 0 |
| Cefepime | 24 | 21 | 5 | 8 |
| Cefotaxime | 20 | 17 | 0 | 2 |
| Cefoxitime | 21 | 24 | 14 | 11 |
| Ceftazidime | 29 | 37 | 2 | 5 |
| Ceftriaxome | 24 | 31 | 1 | 0 |
| Cefuroxime | 8 | 17 | 0 | 0 |
| Chloramphenicol | 0 | 0 | 1 | 3 |
| Chloramphenicol (30 µg) | 1 | 2 | 0 | 0 |
| Ciprofloxacin | 30 | 28 | 12 | 18 |
| Clindamycin | 0 | 0 | 2 | 2 |
| Clindamycin | 5 | 9 | 0 | 0 |
| Ertapenem | 9 | 11 | 0 | 0 |
| Erythromyin | 6 | 9 | 12 | 17 |
| Fosfomycin | 0 | 0 | 2 | 0 |
| Fusidic Acid | 2 | 7 | 0 | 0 |
| Gentomycin | 25 | 23 | 7 | 11 |
| Impenem | 14 | 13 | 0 | 0 |
| Levofloxacin | 0 | 0 | 0 | 0 |
| Linezolid | 0 | 0 | 1 | 0 |
| Meropenem | 17 | 14 | 0 | 0 |
| Moxifloxacin | 0 | 0 | 0 | 0 |
| Oxacillin | 0 | 0 | 0 | 3 |
| Penicillin | 7 | 7 | 7 | 11 |
| Piperacillin | 0 | 0 | 1 | 0 |
| Piperacillin + tazobactam | 16 | 20 | 1 | 2 |
| Quinupristin/dalfopristin | 0 | 0 | 1 | 1 |
| Rifampicin | 3 | 3 | 0 | 0 |
| Sulbactam/Ampicillin | 0 | 0 | 2 | 2 |
| Tetracycline | 1 | 1 | 10 | 6 |
| Tobramycin | 22 | 18 | 0 | 0 |
| Trimethoprim/sulfamethoxazole | 20 | 26 | 4 | 7 |
| Vancomycin | 0 | 1 | 0 | 0 |

Table S7. Number of isolates from each ward showing phenotypic resistances to each antibiotic tested.
