# Supplementary material for: A pandemic within a pandemic? Admission to COVID-19 wards in hospitals is associated with increased prevalence of antimicrobial resistance in two African settings
Source: Ann Clin Microbiol Antimicrob. 2023 Apr 13;22:25. doi: 10.1186/s12941-023-00575-1 (PMC10101537; doi:10.1186/s12941-023-00575-1)
Supplement: Supplementary file 8 — Supplementary Table S8: Antibiotic resistance genes identified in Gram-negatives this study. The heat map shows the percentage of isolates sequenced on each ward that had at least one version of the gene present [file 12941_2023_575_MOESM8_ESM.docx]

|  |  |  | **Sudan** | | **Zambia** | |
| --- | --- | --- | --- | --- | --- | --- |
| **Class** | **Gene** | **Total no. genes identified** | **non-COVID-19 ward** | **COVID-19 ward** | **non-COVID-19 ward** | **COVID-19 ward** |
| β lactam | *bla*ACT | 2 | 0% | 0% | 0% | 17% |
|  | *bla*ADC | 1 | 0% | 0% | 0% | 33% |
|  | *bla*CARB | 5 | 0% | 0% | 0% | 33% |
|  | *bla*KPC | 5 | 100% | 77% | 20% | 0% |
|  | *bla*SHV | 29 | 91% | 100% | 60% | 17% |
|  | *bla*CMY | 24 | 0% | 0% | 40% | 0% |
|  | *bla*TEM | 21 | 91% | 92% | 60% | 33% |
|  | *bla*CTX-M | 23 | 100% | 92% | 80% | 33% |
|  | *bla*OXA | 5 | 9% | 8% | 40% | 67% |
|  | *bla*NDM | 4 | 0% | 8% | 40% | 17% |
|  | *bla*VIM | 2 | 0% | 0% | 20% | 0% |
| Amino. and fluoro. | *aadA* | 8 | 100% | 85% | 60% | 33% |
|  | *aac* | 15 | 100% | 85% | 100% | 100% |
|  | *armA* | 1 | 0% | 0% | 20% | 0% |
|  | *aph* | 12 | 91% | 77% | 80% | 67% |
|  | *ant* | 7 | 0% | 0% | 0% | 33% |
|  | *oqa* | 2 | 0% | 8% | 60% | 33% |
|  | *rmt* | 2 | 91% | 77% | 20% | 0% |
| Other | *sul* | 23 | 91% | 85% | 100% | 67% |
|  | ARR | 3 | 0% | 0% | 20% | 0% |
|  | *cat* | 2 | 0% | 0% | 40% | 0% |
|  | *cml* | 1 | 0% | 0% | 20% | 0% |
|  | *dfr* | 18 | 82% | 62% | 80% | 100% |
|  | *ere* | 1 | 0% | 0% | 20% | 0% |
|  | *erm* | 4 | 0% | 0% | 20% | 17% |
|  | *isr* | 3 | 0% | 0% | 0% | 17% |
|  | *msr* | 1 | 0% | 0% | 20% | 50% |
|  | *mph* | 5 | 0% | 8% | 40% | 50% |
|  | *mdf* | 2 | 9% | 54% | 0% | 0% |
|  | *tet* | 10 | 100% | 77% | 60% | 67% |
|  | *fos* | 5 | 100% | 92% | 60% | 33% |
|  | *qnr* | 7 | 0% | 8% | 60% | 33% |
|  | *qac* | 1 | 73% | 85% | 100% | 17% |
|  | *flo* | 2 | 91% | 77% | 0% | 0% |
|  | *sit* | 1 | 0% | 8% | 20% | 0% |

Table S8. Antibiotic resistance genes identified in Gram-negatives this study. The heat map shows the percentage of isolates sequenced on each ward that had at least one version of the gene present.
