# Supplementary material for: A pandemic within a pandemic? Admission to COVID-19 wards in hospitals is associated with increased prevalence of antimicrobial resistance in two African settings
Source: Ann Clin Microbiol Antimicrob. 2023 Apr 13;22:25. doi: 10.1186/s12941-023-00575-1 (PMC10101537; doi:10.1186/s12941-023-00575-1)
Supplement: Supplementary file 9 — Supplementary Table S9: Number of plasmids found on each ward and the percentage of isolates on each ward that they were identified in [file 12941_2023_575_MOESM9_ESM.docx]

|  | **Sudan** | | **Zambia** | |
| --- | --- | --- | --- | --- |
|  | non-COVID-19 ward | COVID-19 ward | non-COVID-19 ward | COVID-19 ward |
| IncC | 10 (100%) | 10 (83%) | 1 (20%) | 0 (0%) |
| IncR | 10 (100%) | 10 (83%) | 1 (20%) | 0 (0%) |
| IncQ1 | 0 (0%) | 0 (0%) | 1 (20%) | 0 (0%) |
| IncFII(29) | 0 (0%) | 0 (0%) | 1 (20%) | 0 (0%) |
| IncFII(K) | 6 (60%) | 9 (75%) | 2 (40%) | 0 (0%) |
| IncFIA(HI1) | 0 (0%) | 0 (0%) | 2 (40%) | 0 (0%) |
| IncFIB(K) | 0 (0%) | 0 (0%) | 1 (20%) | 0 (0%) |
| IncFIB(pKPHS1) | 8 (80%) | 11 (92%) | 1 (20%) | 0 (0%) |
| IncFIB(pQil) | 0 (0%) | 0 (0%) | 1 (20%) | 0 (0%) |
| IncFIB(pNDM-Mar) | 0 (0%) | 0 (0%) | 1 (20%) | 0 (0%) |
| IncHI1B(pNDM-MAR) | 0 (0%) | 0 (0%) | 1 (20%) | 0 (0%) |
| FIA(pBK30683) | 0 (0%) | 1 (8%) | 0 (0%) | 0 (0%) |
| Col440I | 0 (0%) | 0 (0%) | 1 (20%) | 0 (0%) |
| Col440II | 0 (0%) | 1 (8%) | 1 (20%) | 0 (0%) |
| ColRNAI | 0 (0%) | 0 (0%) | 0 (0%) | 1 (17%) |
| Col3M | 0 (0%) | 0 (0%) | 1 (20%) | 0 (0%) |
| **Total plasmids found** | **4** | **6** | **14** | **1** |

Table S9. Number of plasmids found on each ward and the percentage of isolates on each ward that they were identified in.
